# Supplementary material for: The chronODE framework for modelling multi-omic time series with ordinary differential equations and machine learning
Source: Nat Commun. 2025 Aug 19;16:7021. doi: 10.1038/s41467-025-61921-9 (PMC12365117; doi:10.1038/s41467-025-61921-9)
Supplement: Supplementary file 1 — Supplementary Information [file 41467_2025_61921_MOESM1_ESM.pdf]

The chronODE framework for modelling multi-omic time series with ordinary differential equations and machine learning

Beatrice Borsari[1,2*], Mor Frank[1,2*], Eve S. Wattenberg[1,2#], Ke Xu[3#], Susanna X. Liu[1,2#], Xuezhu Yu[1,2], and Mark Gerstein[1,2,3,4,5†]

[1]Program in Computational Biology and Biomedical Informatics, Yale University, New Haven, CT, 06511, USA
[2]Department of Molecular Biophysics and Biochemistry, Yale University, New Haven, CT, 06511, USA
[3]Department of Computer Science, Yale University, New Haven, CT, 06511, USA
[4]Department of Statistics and Data Science, Yale University, New Haven, CT, 06511, USA
[5]Department of Biomedical Informatics and Data Science, Yale University, New Haven, CT, 06511, USA
[*]Equally contributing authors
[#]Equally contributing authors
[†]Corresponding author: mark@gersteinlab.org

Supplementary Information

Supplementary Figures 1-8
Supplementary Tables 1-3
Supplementary Note 1

Supplementary Figure 1

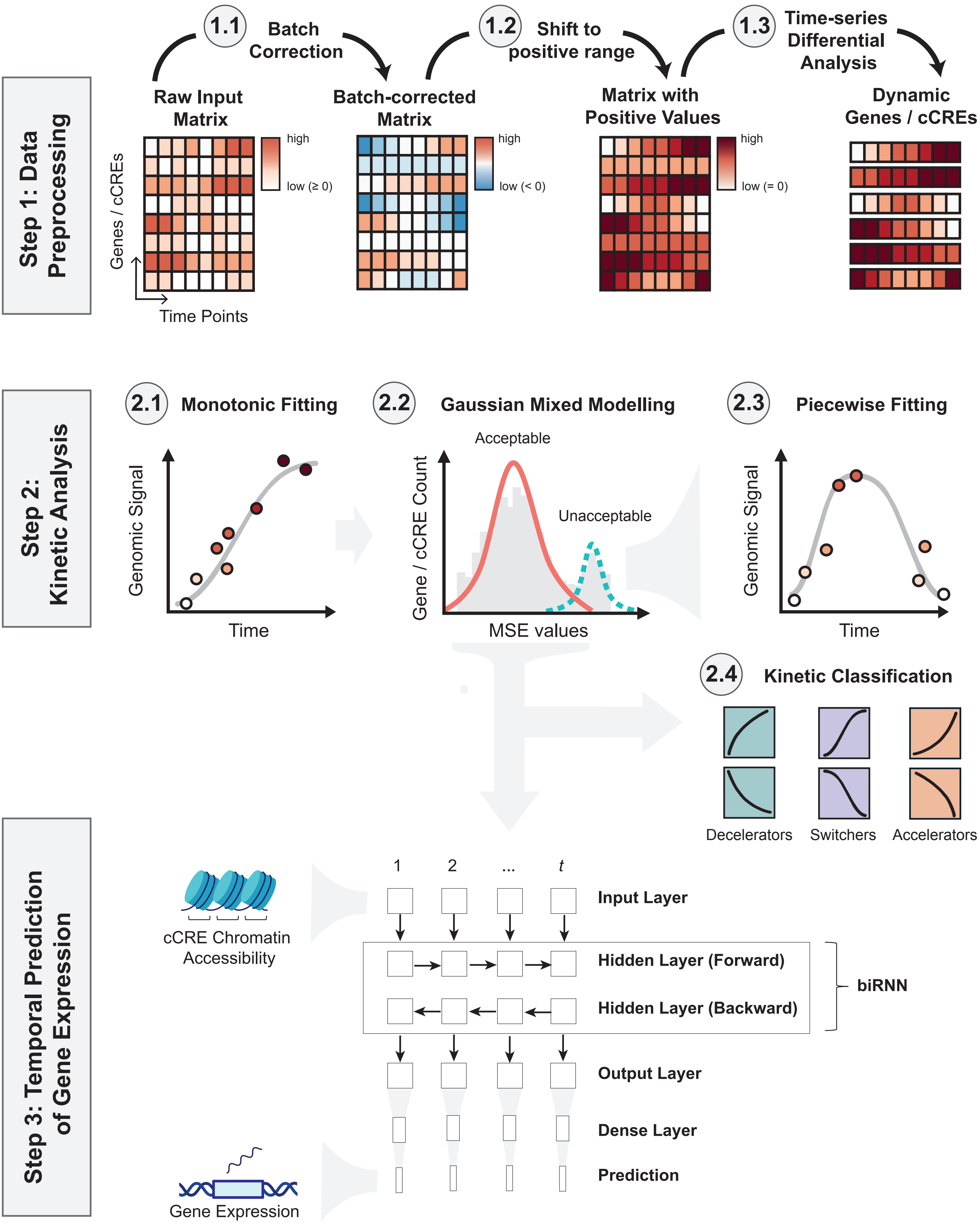

Overview of the chronODE modeling framework. The framework processes time-series signals for genes or cCREs through three main steps: Data Preprocessing, Kinetic Analysis, and Temporal Prediction of Gene Expression. In the first step, it performs batch correction, shifts values to a positive range, and conducts time-series differential analysis. Next, each time-series profile undergoes monotonic fitting, and the quality of the fit is assessed by partitioning the distribution of Mean Squared Errors (MSE) into acceptable (red) and unacceptable (cyan) fits using Gaussian Mixed Modelling (see also Supplementary Fig. 3D). Profiles with acceptable fits are then classified into three kinetic classes: switchers, accelerators, and decelerators. Unacceptable fits are instead modeled using a piecewise approach to better capture alternative kinetic patterns, such as peak-like profiles that are not well described by monotonic functions. Finally, the monotonic-fitted cCRE trajectories serve as input to a bidirectional neural network (biRNN), which predicts the temporal expression of putative target genes. Elements of this figure were created in BioRender. Gerstein, M. (2025) `https://BioRender.com/837r9aa`.

Supplementary Figure 2

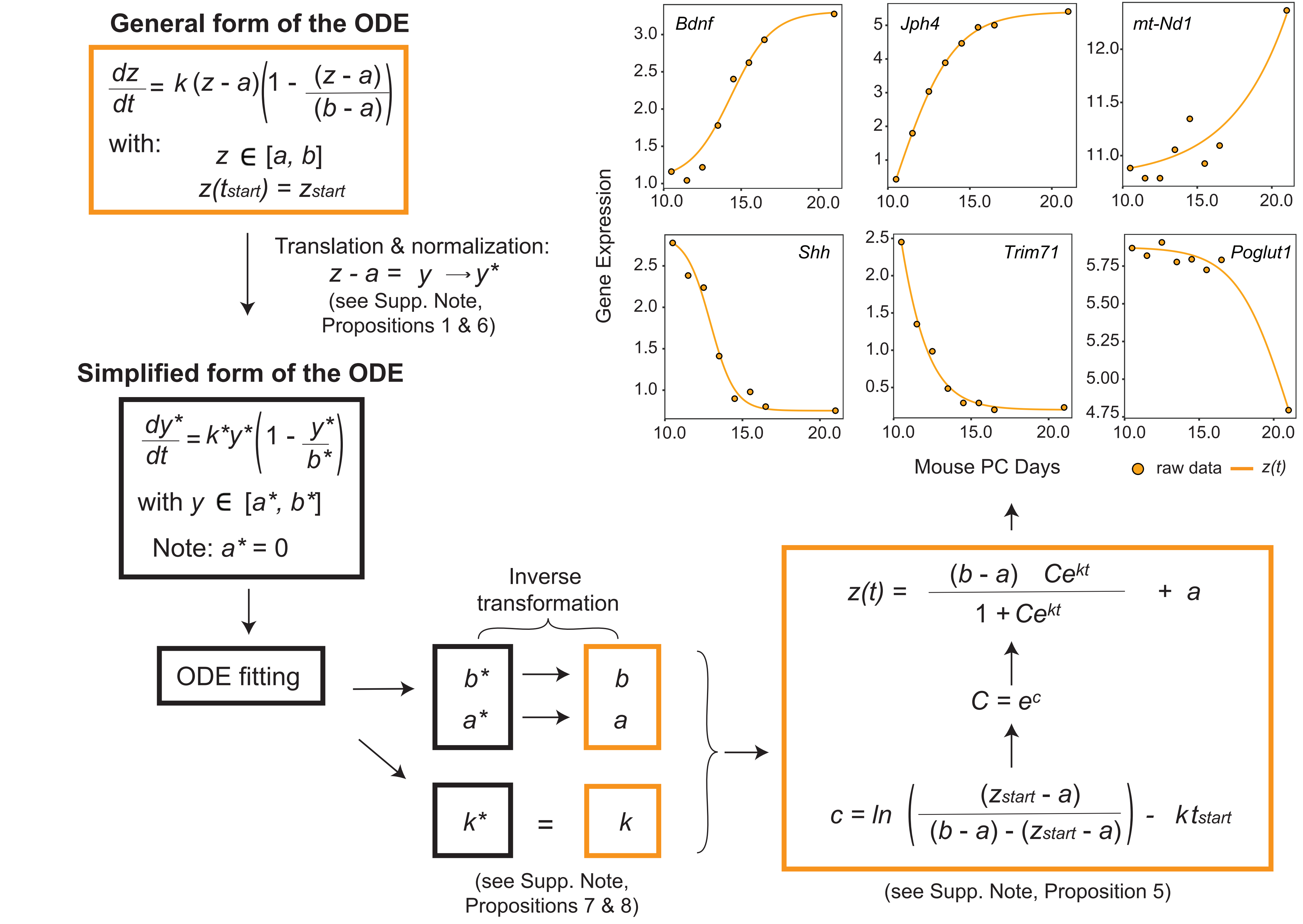

Supplementary Figure 2

Transformation and fitting of a generalized logistic ODE function to model time-series functional genomics data. Top left: The general form of the ODE (Eq. 1) describing time-series gene expression or chromatin signal $z(t)$ is parametrized by three parameters (the rate constant k, and the lower and upper asymptotes a and b), and follows a logistic-like curve. Middle left: This general form is transformed into a simplified ODE with two parameters (k^* and b^*, assuming a lower asymptote $a^* = 0$). The time-series signal y^* is obtained upon min-max normalization of $z(t)$ (see Methods); y^*_{start} corresponds to the normalized value y^* measured at time point t_{start} (i.e., the first time point of the experimental time course). We numerically estimate the parameters k^* and b^* that best fit the data. In Propositions 7-8 (Supplementary Note 1), we show that both translation and normalization of the data from $z(t)$ to y^* preserve the value of k as it would appear in the real (observed) data range. On the other hand, a and b in the real range can be recovered using an inverse transformation (see Methods, "Restoring the fitted curve to the original range of the data"). Bottom right: The analytical solution $z(t)$ is directly derived using these parameters, allowing reconstruction of the smooth time-series functional genomics data trajectories, demonstrating that the parameters k^* and b^* from the simplified ODE can be interpreted in the context of the original data. Right: Examples of model fits (orange curves) to raw gene expression data (black dots). Note that these fits are the same shown in Fig. 2C.

Supplementary Figure 3

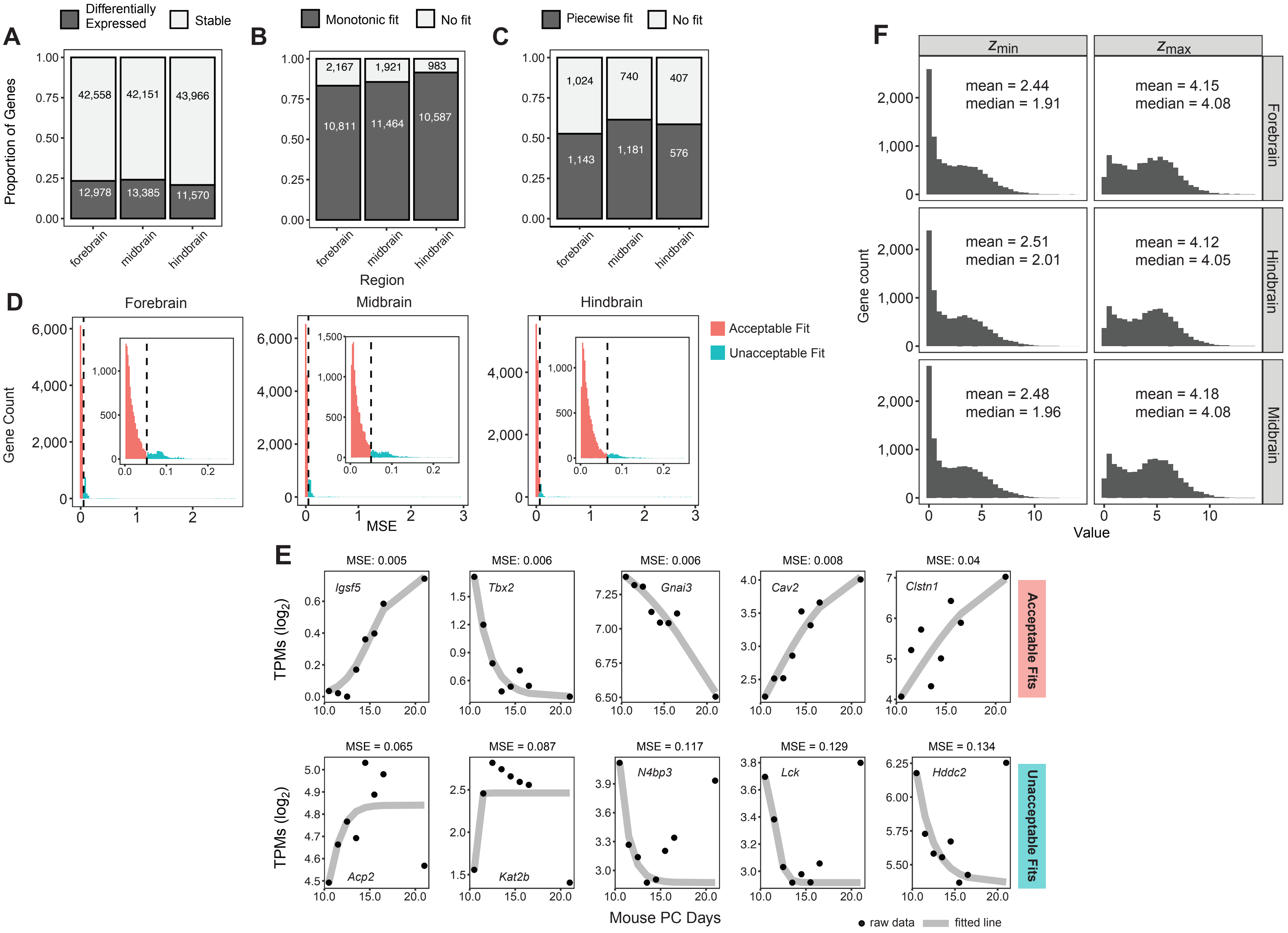

Supplementary Figure 3

Monotonic and piecewise fitting of gene expression kinetics during mouse brain development. **A**: Proportion of genes (y axis) that are stably or differentially expressed over time across the three regions (x axis). **B-C**: Analogous representation to panel A for genes with monotonic and piecewise fits. **D**: Distribution of Mean Squared Error (MSE) of monotonically fitted genes across the three brain regions. The distribution is partitioned into acceptable (red) and unacceptable (cyan) fits following Gaussian Mixed Model (GMM) analysis. The GMM cutoff is indicated by a dashed vertical line. Forebrain: GMM cutoff = 0.053 (*n* = 12,286; 2 biological replicates); midbrain: GMM cutoff = 0.050 (*n* = 13,038; 2 biological replicates); hindbrain: GMM cutoff = 0.065 (*n* = 11,341; 2 biological replicates). **E**: Examples of acceptable and unacceptable monotonic fits with the corresponding MSE values. The x axis represents mouse post-conception (PC) days, and the y axis shows gene expression values expressed in Transcripts Per Million (TPMs, $\log_2$-transformed). The black dots correspond to the data points prior to monotonic fitting, and the grey line corresponds to the monotonically fitted expression profile. **F**: Distributions of the scaling factors z_{min} and z_{max} employed in the min-max normalization from z to y^* (see Methods section “Data normalization”) for gene expression data across the three brain regions (forebrain: *n* = 10,811, 2 biological replicates; midbrain: *n* = 11,464, 2 biological replicates; hindbrain: *n* = 10,587, 2 biological replicates).

Supplementary Figure 4

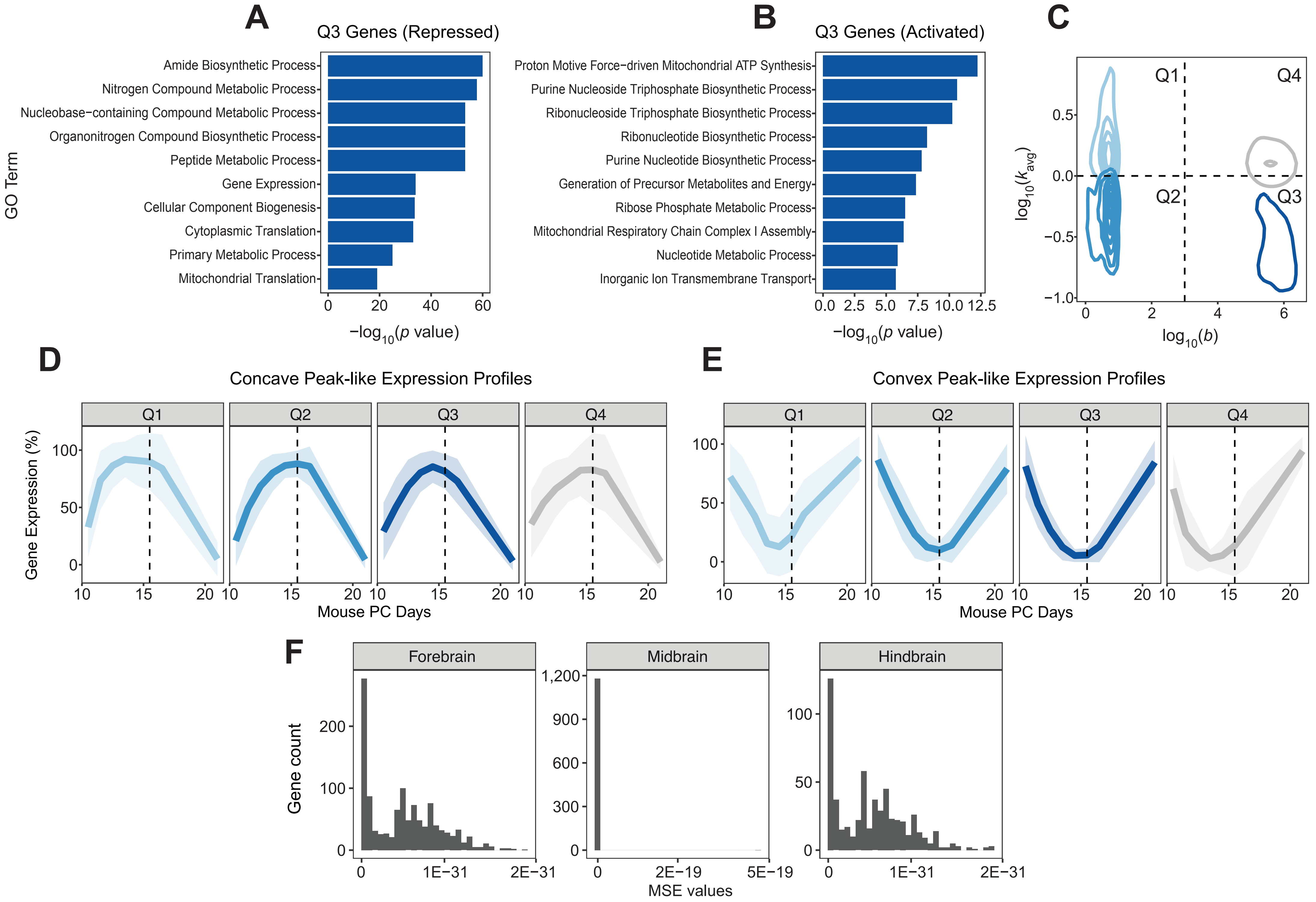

Supplementary Figure 4

Properties of Q1-Q4 genes. A-B: Gene Ontology biological process terms (y axis) enriched among repressed and activated Q3 monotonic genes with the corresponding -$\log_{10}$ *p* value (x axis). Significance of GO terms' enrichment was determined with a hypergeometric test (two-sided). Only GO terms reporting a Benjamini-Hochberg adjusted *p* value < 0.01 were considered. Among these, we show in the panel the 10 most significant GO terms. **C**: Distribution of the kinetic parameters k (magnitude expressed in absolute value, y axis) and b (x axis) across all genes with piecewise sigmoid fits in the three brain regions. For genes with piecewise fits, two k values are computed (k_{left} and k_{right}, one for each sigmoid segment) and here we display their average absolute k value (k_{avg}). The plot subdivision in four Q1-Q4 quadrants is the same shown in Fig. 2A. **D-E**: Lineplot showing, for genes modelled by piecewise fits in Q1 through Q4, the average expression (y axis) over time (x axis; PC = Post-Conception). Expression profiles with concave peaks are characterized by $k_{\text{left}} > 0$ (**D**), while those with convex peaks have $k_{\text{left}} < 0$ (**E**). Note that the average gene expression levels (expressed in $\log_2$-transformed TPMs) were rescaled to the range 0-100% to allow for comparison across Q1-Q4 groups. The vertical dashed line indicates day E15.5 (middle point of the time-course). The error band corresponds to the standard deviation of the mean rescaled expression level (i.e., mean±SD). **F**: Distributions of Mean Squared Error (MSE) values computed between the ODE-based piecewise fitting and the B-spline piecewise fitting for genes exhibiting peak-like expression patterns across the three brain regions (forebrain: *n* = 1,143; midbrain: *n* = 1,181; hindbrain: *n* = 576).

Supplementary Figure 5

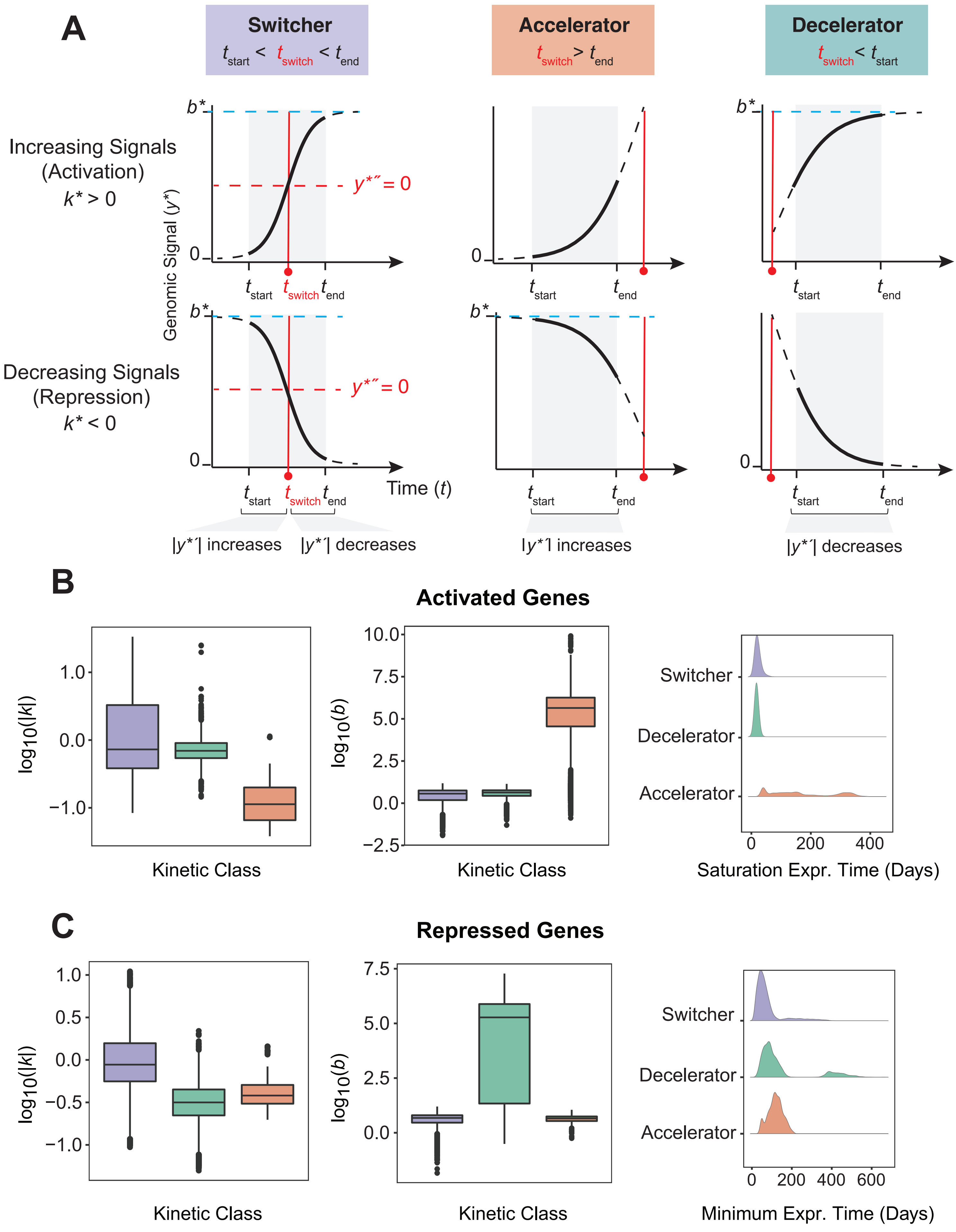

Supplementary Figure 5

Kinetic classification of monotonic fits. **A**: Schematic of monotonic logistic curves modeled by chronODE for increasing (upper panel) and decreasing (lower panel) signals, using the simplified form of the ODE (see also Supplementary Fig. 2). Dashed horizontal blue lines: saturation point (b^*). Solid vertical red lines: t_{switch} such that $y^{*\prime\prime}(t_{\text{switch}}) = 0$. Dashed red lines: $y^*(t_{\text{switch}})$. Switcher curves include both positive and negative $y^{*\prime\prime}$ because t_{switch} lies within the time frame of interest defined by t_{start} and t_{end}. Accelerator curves do not reach t_{switch} until after t_{end}. For decelerator curves, t_{switch} occurs before t_{start}. **B**: Distribution of $|k|$, b, and time point of expression saturation for activated genes across the three kinetic classes (accelerator, decelerator, switcher; color-coded as in panel A). Box plots present the median as the center, 25% and 75% percentiles as box limits, and whiskers extending to the largest and smallest values within the 1.5 × IQR of the box limits (*n* genes = 19,957). **C**: Distribution of $|k|$, b, and time point of minimum expression for repressed genes across the three kinetic classes (*n* genes = 12,905). Panels B and C summarize information across the three brain regions.

Supplementary Figure 6

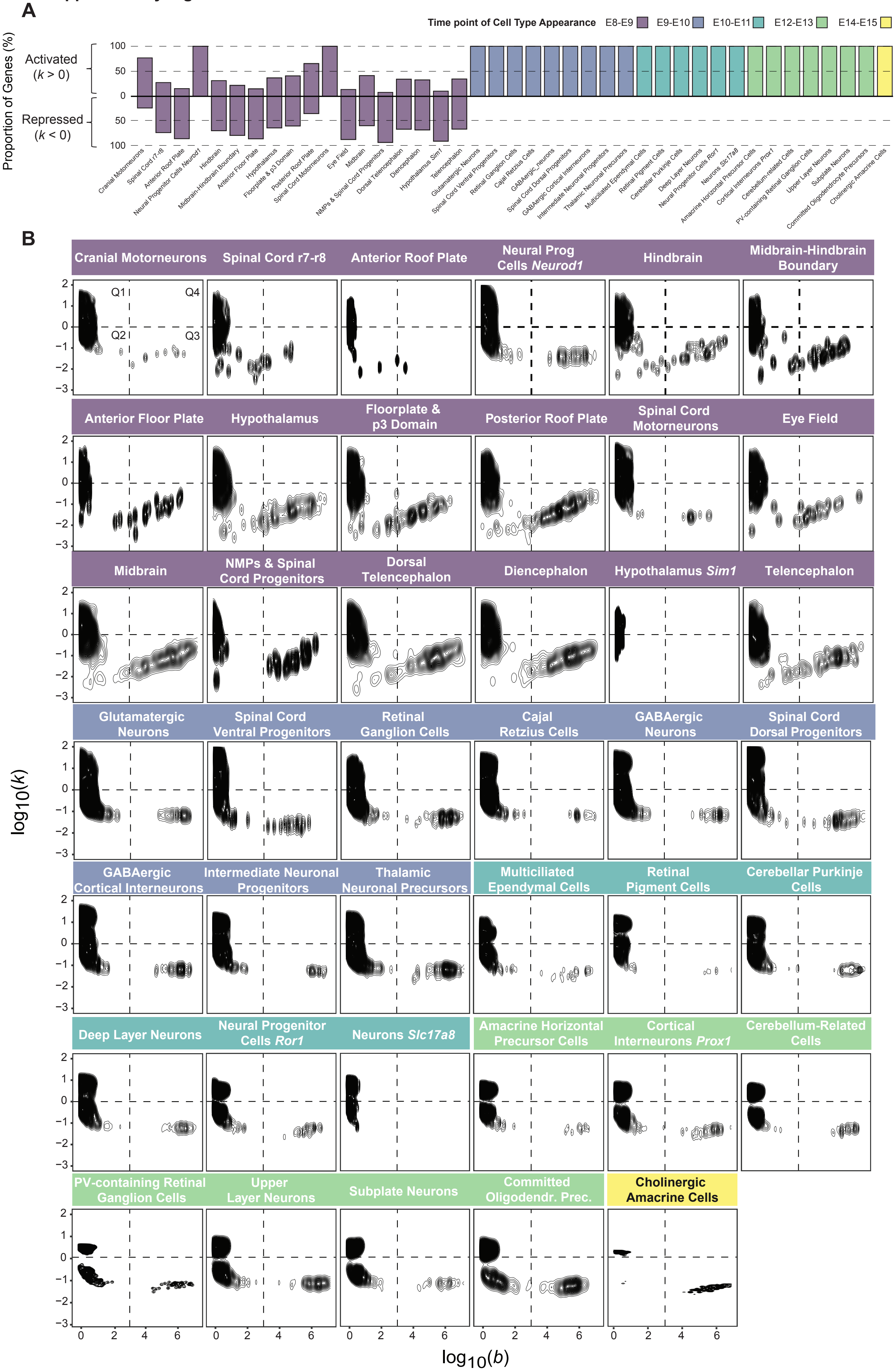

Supplementary Figure 6

Analysis of gene expression kinetics at single-cell resolution. **A**: Proportion (y axis) of activated ($k > 0$) and repressed ($k < 0$) genes with acceptable monotonic fits across 41 brain cell types (x axis). Cell types are color-coded according to the time point of appearance. **B**: Distribution of the kinetic parameters k (y axis) and b (x axis) for monotonically fitted genes expressed in each cell type. Only activated genes are shown. The plot subdivision in four Q1-Q4 quadrants is the same shown in Fig. 2A.

Supplementary Figure 7

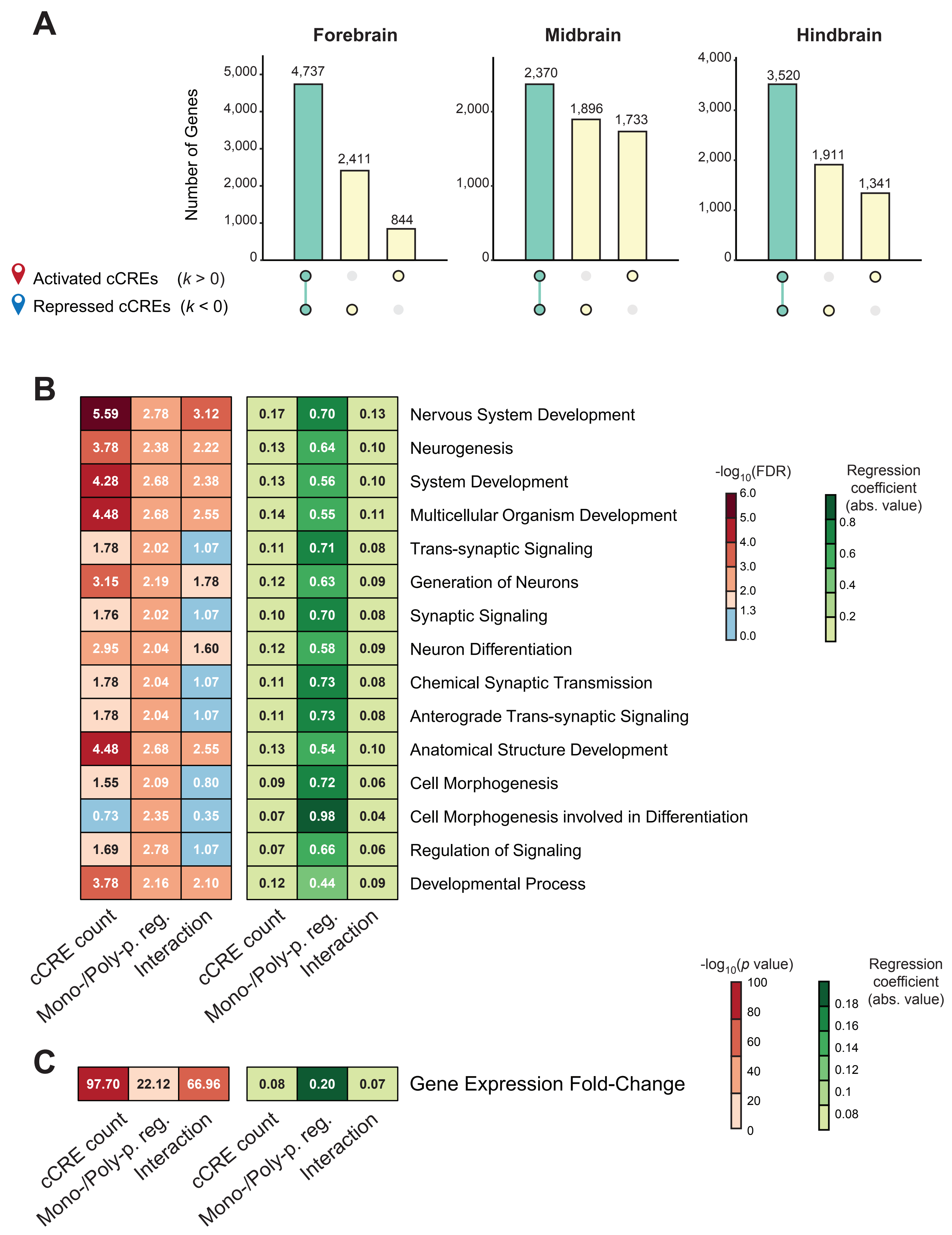

Supplementary Figure 7

Properties of mono- and poly-pattern genes. **A**: Upset plots showing, for each mouse brain region, the number of genes (y axis) that are associated with activated ($k > 0$) and repressed ($k < 0$) cCREs. Mono-, and poly-pattern genes are associated with one (yellow) and two (turquoise) types of cCREs, respectively (see also Fig. 4A). **B**: Logistic regression analysis of gene-GO term associations. For each of the 15 GO terms most significantly associated with poly-pattern genes (see Fig. 4D), we performed the regression analysis using a generalized linear model, specifying family = binomial and link = logit. The left heatmap shows the -$\log_{10}$ False Discovery Rate (FDR)-adjusted p values for the cCRE count coefficient, the poly-pattern coefficient, and their interaction term. The right heatmap displays the corresponding regression coefficients (expressed as absolute values) for each term. For more details, see Methods section “Mono-pattern & poly-pattern genes”. **C**: Analogous representation for the results obtained through a linear model estimating the effect of cCRE count and poly-pattern regulation (independent variables) on gene expression fold-change (dependent variable).

Supplementary Figure 8

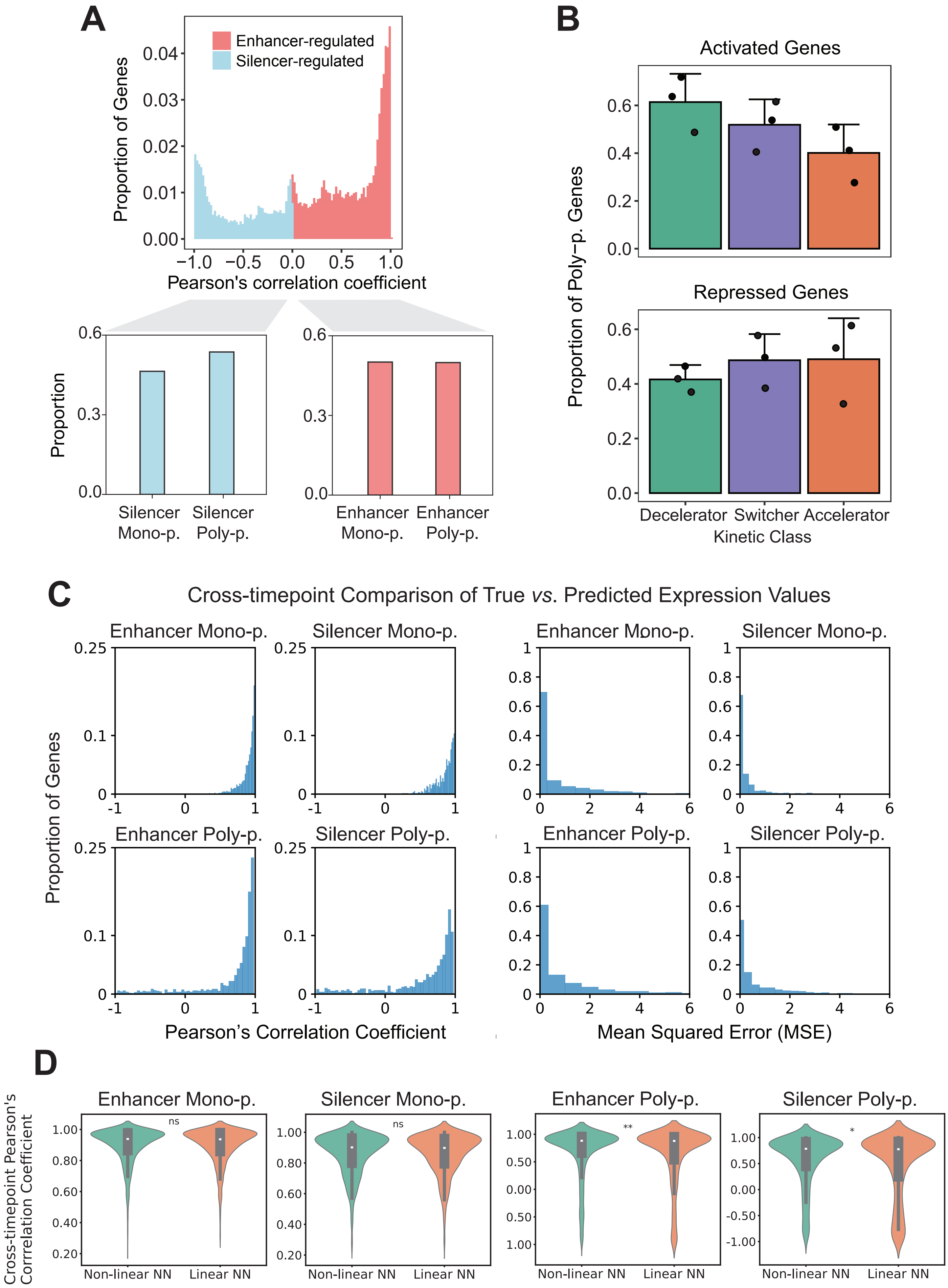

Supplementary Figure 8

biRNN predictions across four gene regulatory mechanisms. A. Upper panel: Distribution of Pearson's correlation coefficients between time-series vectors of cCRE chromatin signals and gene expression signals across the three brain regions. For each gene, we computed the average correlation across all associated cCREs. Genes were then classified into two groups based on the type of cCRE regulation: enhancer-regulated (Pearson correlation coefficient > 0, red) or silencer-regulated (Pearson correlation coefficient < 0, lightblue). **Lower panel**: Within each regulatory category (enhancer or silencer), we further report the proportion of genes exhibiting mono-pattern (mono-p.) or poly-pattern (poly-p.) regulation. **B**: Proportion of poly-pattern genes (y axis) among genes characterized by decelerator, switcher, and accelerator expression profiles. Upper and lower panel distinguish between activated $(k > 0)$ and repressed $(k < 0)$ genes. We report mean proportion and standard deviation across the three brain regions (*n* = 3). Overlayed black dots indicate the proportion of genes in each region. **C. Left panel**: Distribution of cross-timepoints Pearson's correlation computed between the true and predicted values for each gene across time points. Mean correlation was computed for each of the four regulatory mechanisms (Enhancer mono-pattern: 0.90, Silencer mono-pattern: 0.86, Enhancer poly-pattern: 0.68, and Silencer poly-pattern: 0.57). **Right panel**: Distributon of cross-timepoints Mean Squared Error (MSE) computed between the true and predicted values for each gene across time points. Mean MSE was computed for each of the four regulatory mechanisms (Enhancer mono-pattern: 0.87, Silencer mono-pattern: 0.39, Enhancer poly-pattern: 1.24, Silencer poly-pattern: 1.02). **D**: Distributions of cross-timepoint Pearson's correlations between true and predicted gene expression values using a linear (orange) or non-linear (green) model. The baseline linear model corresponds to a neural network (NN) without bidirectional RNN (biRNN) and ReLU activation. Statistically significant differences between distributions were assessed using the Wilcoxon Rank-Sum test (two-sided, paired). ns = not significant (*p* value > 0.05); *: *p* value < 0.05; **: *p* value < 0.01. For poly-pattern genes, the linear NN shows significantly lower correlations than the non-linear NN (enhancer poly-pattern: *p* value = 0.0043; silencer poly-pattern: *p* value = 0.0285). No significant differences were observed for mono-pattern genes (enhancer mono-pattern: *p* value = 0.736; silencer mono-pattern: *p* value = 0.2227). Box plots present the median as the center, 25% and 75% percentiles as box limits, and whiskers extending to the largest and smallest values within the 1.5 × IQR of the box limits.

Supplementary Tables

Region	Time point	Experiment ID	Quantification tsv file ID
Forebrain	E10.5 days	ENCSR304RDL	ENCFF476ADM, ENCFF145PTV
Midbrain	E10.5 days	ENCSR764OPZ	ENCFF875HME, ENCFF126VCW
Hindbrain	E10.5 days	ENCSR943LKA	ENCFF169CJH, ENCFF997ZPU
Forebrain	E11.5 days	ENCSR160IIN	ENCFF696BTU, ENCFF042VCB
Midbrain	E11.5 days	ENCSR307BCA	ENCFF094JLI, ENCFF184FWR
Hindbrain	E11.5 days	ENCSR760TOE	ENCFF606UHO, ENCFF434CSI
Forebrain	E12.5 days	ENCSR647QBV	ENCFF698XIB, ENCFF649WEQ
Midbrain	E12.5 days	ENCSR908JWT	ENCFF521YOL, ENCFF840AXS
Hindbrain	E12.5 days	ENCSR420QTO	ENCFF928MQD, ENCFF046RSQ
Forebrain	E13.5 days	ENCSR970EWM	ENCFF794PWS, ENCFF088OEQ
Midbrain	E13.5 days	ENCSR792RJV	ENCFF867NEL, ENCFF189APQ
Hindbrain	E13.5 days	ENCSR921PRX	ENCFF960KJV, ENCFF356CTG
Forebrain	E14.5 days	ENCSR185LWM	ENCFF270DCV, ENCFF565AWX
Midbrain	E14.5 days	ENCSR343YLB	ENCFF743IEH, ENCFF954BEO
Hindbrain	E14.5 days	ENCSR559TRB	ENCFF304ILZ, ENCFF876LKY
Forebrain	E15.5 days	ENCSR752RGN	ENCFF080PBH, ENCFF890FJF
Midbrain	E15.5 days	ENCSR557RMA	ENCFF670AQP, ENCFF624EQM
Hindbrain	E15.5 days	ENCSR401BSG	ENCFF195CMT, ENCFF804NPY
Forebrain	E16.5 days	ENCSR080EVZ	ENCFF719ADL, ENCFF029UVS
Midbrain	E16.5 days	ENCSR367ZPZ	ENCFF238YUA, ENCFF762ZJZ
Hindbrain	E16.5 days	ENCSR285WZV	ENCFF310GSV, ENCFF858ZON
Forebrain	1st PN day	ENCSR362AIZ	ENCFF484FBW, ENCFF143OBR
Midbrain	1st PN day	ENCSR719NAJ	ENCFF492ODZ, ENCFF532MRQ
Hindbrain	1st PN day	ENCSR017JEG	ENCFF892WXB, ENCFF851KEG

Supplementary Table 1. Experiment and file identifiers for mouse RNA-seq experiments obtained from the ENCODE portal (`https://www.encodeproject.org/`).

Region	Time point	Experiment ID	bigBed file ID	bigWig file ID
Forebrain	E10.5 days	ENCSR756SPS	ENCFF880ICH, ENCFF316KAS, ENCFF287EYQ, ENCFF544FGH	ENCFF506ZSC, ENCFF044ISO
Midbrain	E10.5 days	ENCSR773SAG	ENCFF181QNC, ENCFF317OFK, ENCFF534WLB, ENCFF321PTE	ENCFF293YCS, ENCFF330OMV
Hindbrain	E10.5 days	ENCSR289BTM	ENCFF096PEE, ENCFF020ZKO, ENCFF862MJH, ENCFF920TRY	ENCFF790CVH, ENCFF072IWN
Forebrain	E11.5 days	ENCSR014SFF	ENCFF050WLI, ENCFF993LQC, ENCFF779DJK, ENCFF271HGS	ENCFF639LUT, ENCFF580FOS
Midbrain	E11.5 days	ENCSR292QBA	ENCFF382EUY, ENCFF524WVA	ENCFF414SMG
Hindbrain	E11.5 days	ENCSR358ESL	ENCFF113JBH, ENCFF656GBX, ENCFF465HTG, ENCFF344ZDH	ENCFF587MWA, ENCFF609ATS
Forebrain	E14.5 days	ENCSR337EDG	ENCFF821UVA, ENCFF854HCQ, ENCFF225UEN, ENCFF093PQD	ENCFF237YNH, ENCFF931CQE
Midbrain	E14.5 days	ENCSR367FCW	ENCFF310VOE, ENCFF876PHQ	ENCFF539CBL
Hindbrain	E14.5 days	ENCSR179PIH	ENCFF702YOI, ENCFF083VZJ, ENCFF858OHC, ENCFF965UXS	ENCFF274NPS, ENCFF537XKO
Forebrain	1st PN day	ENCSR791AJY	ENCFF217LRD, ENCFF928NJG	ENCFF727CYI
Midbrain	1st PN day	ENCSR767AJS	ENCFF305HSJ, ENCFF125JIF, ENCFF277HPA, ENCFF650WJJ	ENCFF325FZS, ENCFF584BTI
Hindbrain	1st PN day	ENCSR469VGZ	ENCFF868GJZ, ENCFF233WJN, ENCFF314FJW, ENCFF500TCE	ENCFF822FDB, ENCFF286LUT

Supplementary Table 2. Experiment and file identifiers for mouse DNase-seq experiments obtained from the ENCODE portal (https://www.encodeproject.org/).

**Region**	**Time point**	**Experiment ID**	**bigBed file ID**	**bigWig file ID**
Forebrain	E11.5 days	ENCSR273UFV	ENCFF582AQM	ENCFF609FLS, ENCFF014TVL
Midbrain	E11.5 days	ENCSR382RUC	ENCFF697ALQ	ENCFF298FKT, ENCFF886SFL
Hindbrain	E11.5 days	ENCSR012YAB	ENCFF693MQK	ENCFF067IQO, ENCFF129DMB
Forebrain	E12.5 days	ENCSR559FAJ	ENCFF724CPC	ENCFF314LIV, ENCFF586FIG
Midbrain	E12.5 days	ENCSR154BXN	ENCFF818SNO	ENCFF460PDT, ENCFF860MZS
Hindbrain	E12.5 days	ENCSR088UYE	ENCFF146WID	ENCFF533PFX, ENCFF642QRZ
Forebrain	E13.5 days	ENCSR903GMO	ENCFF761PYJ	ENCFF278ROE, ENCFF161RRE
Midbrain	E13.5 days	ENCSR819QOJ	ENCFF490UUC	ENCFF063FOL, ENCFF355SPQ
Hindbrain	E13.5 days	ENCSR176BYZ	ENCFF894TOH	ENCFF953AOC, ENCFF280BOI
Forebrain	E14.5 days	ENCSR810HQR	ENCFF197DQD	ENCFF092VLN, ENCFF039BNT
Midbrain	E14.5 days	ENCSR384JBF	ENCFF019TUF	ENCFF927RDV, ENCFF518JWO
Hindbrain	E14.5 days	ENCSR798FDL	ENCFF859DRE	ENCFF572TQP, ENCFF823LHI
Forebrain	E15.5 days	ENCSR976LWP	ENCFF501WGN	ENCFF398CBN, ENCFF764JRZ
Midbrain	E15.5 days	ENCSR468GUI	ENCFF821IXB	ENCFF356BYP, ENCFF106YOY
Hindbrain	E15.5 days	ENCSR662KNY	ENCFF748XVF	ENCFF566SYW, ENCFF985BES
Forebrain	E16.5 days	ENCSR836PUC	ENCFF779DKA	ENCFF365KJF, ENCFF807OVL
Midbrain	E16.5 days	ENCSR096JCC	ENCFF755XMC	ENCFF662ETT, ENCFF074YXW
Hindbrain	E16.5 days	ENCSR623GSD	ENCFF586TSG	ENCFF770SZX, ENCFF800APZ
Forebrain	1st PN day	ENCSR310MLB	ENCFF178FIA	ENCFF171GQJ, ENCFF374YJI
Midbrain	1st PN day	ENCSR211OCS	ENCFF750OEN	ENCFF116OYR, ENCFF231JIR
Hindbrain	1st PN day	ENCSR312LQX	ENCFF855NTE	ENCFF981VLS, ENCFF752FVM

Supplementary Table 3. Experiment and file identifiers for mouse ATAC-seq experiments obtained from the ENCODE portal (`https://www.encodeproject.org/`).

Supplementary Note 1

Proposition 1. *The time-series signal of a given gene or cCRE is modeled using the following generalized logistic ordinary differential equation (ODE):*

$$\frac{dz}{dt} = k(z-a)(1-\frac{z-a}{b-a})$$

Here, $z(t)$ denotes the gene or cCRE signal at time t, constrained within the interval $[a,b]$. The parameters a and b correspond to the lower and upper asymptotes of the logistic curve, respectively. k is a rate parameter that determines the steepness of the curve. Let $z_{start} = z(t_{start})$ denote the observed signal at the first time point monitored by the time-course experiment.

*The analytical solution of this ODE is (see **Proposition 5**):*

$$z(t) = \frac{(b-a)Ce^{kt}}{1+Ce^{kt}} + a.$$

However, fitting this general form directly, although more flexible, can lead to potential numerical instability, In fact, with only eight time points (like in the case of the bulk data used in this paper), estimating the three parameters a, b, and k becomes unstable. In other words, the model with three parameters exhibits a higher susceptibility to large convergence errors during parameter fitting, likely due to the increased complexity and flexibility of the parameter space (see also Motulsky & Christopoulos, 2004, Chapter A "Fitting data with non-linear regression")[1].

To mitigate this issue, we consider the simplified form of the logistic ODE:

$$\frac{dy^*}{dt} = k^*y^*(1-\frac{y^*}{b^*}).$$

Note that this equation is a translated version of the generalized form via shifting relative to a (i.e., $z-a=y$), which simplifies the algebraic expression. This allows to reduce the model from three to two parameters, fixing the lower asymptote to zero (i.e., $a=0$) and improving numerical stability while essentially preserving the kinetic features. In this way, we can determine the rate constant k with far more precision[1].

*However, knowing y requires prior knowledge of a. To circumvent this dependency, we instead fit the simplified ODE on a normalized version of y (e.g., y^*), which is equivalent to the normalized version of z (see **Proposition 6** and **Supplementary Fig. 2**). In fact, the normalized data y^* better meets the assumption of the ODE of having a lower asymptote equal to zero (i.e., $a^*=0$).*

We show that the solution for:

$$\frac{dy^*}{dt} = k^*y^*(1-\frac{y^*}{b^*}),$$

where $y^* \in [0,b^*]$ (that is $b^* > y^* > 0$), and $y^*|_{t=t_{start}} = y^*_{start}$, is:

$$y^*(t) = \frac{b^*C^*e^{k^*t}}{b^*+C^*e^{k^*t}}.$$

Proof.

$$\frac{dy^*}{dt} = k^*y^*(1-\frac{y^*}{b^*})$$

$$\frac{dy^*}{y^*(1-\frac{y^*}{b^*})} = k^*dt$$

$$(\frac{1}{y^*} + \frac{\frac{1}{b^*}}{(1-\frac{y^*}{b^*})})dy^* = k^*dt$$

$$ln(y^*) - ln(1-\frac{y^*}{b^*}) = k^*t + c^*,$$

Then, we have:

$$ln(y^*_{\text{start}}) - ln(1-\frac{y^*_{\text{start}}}{b^*}) = k^*t_{\text{start}} + c^*$$

$$c^* = ln(y^*_{\text{start}}) - ln(1-\frac{y^*_{\text{start}}}{b^*}) - k^*t_{\text{start}}$$

$$ln(y^*) - ln(1 - \frac{y^*}{b^*}) = k^*t + c^*$$
$$\frac{y^*}{(1 - \frac{y^*}{b^*})} = C^*e^{k^*t}$$
$$y^* = \frac{b^*C^*e^{k^*t}}{b^* + C^*e^{k^*t}}$$

where $C^* = e^{c^*}$.

Actually, as $(t_{\text{start}}, y^*_{\text{start}})$ is on this line, we have:

$$\frac{y^*_{\text{start}}}{(1 - \frac{y^*_{\text{start}}}{b^*})} = C^*e^{k^*t_{\text{start}}}$$
$$C^* = \frac{y^*_{\text{start}}}{(1 - \frac{y^*_{\text{start}}}{b^*})e^{k^*t_{\text{start}}}}.$$

Therefore, we will get another form of C^* based on the initial time and the fitted parameters. □

Proposition 2. *Given*

$$y^*(t) = \frac{b^*C^*e^{k^*t}}{b^* + C^*e^{k^*t}}$$

we have:

$$\lim_{t\to\infty} y^*(t) = \begin{cases} 0 & \textit{if } k^* < 0 \\ b^* & \textit{if } k^* > 0 \end{cases}$$

Proof. Notice:

$$\begin{aligned} \frac{1}{y^*(t)} &= \frac{b^* + C^*e^{k^*t}}{b^*C^*e^{k^*t}} \\ &= \frac{1}{C^*e^{k^*t}} + \frac{1}{b^*}. \end{aligned}$$

When $k^* > 0$, we have:

$$C^*e^{k^*t} \to \infty$$

when $t \to \infty$.

So:

$$\lim_{t\to\infty} \frac{1}{y^*(t)} = \frac{1}{b^*}$$
$$\lim_{t\to\infty} y^*(t) = b^*.$$

When $k^* < 0$, we have:

$$C^*e^{k^*t} \to 0$$

when $t \to \infty$.

So:

$$\lim_{t\to\infty} \frac{1}{y^*(t)} = \infty$$
$$\lim_{t\to\infty} y^*(t) = 0.$$

□

Proposition 3. *When the switch time is defined to be the time point where the second derivative is 0, we have:*

$$\begin{aligned} t_{switch} &= \frac{ln(\frac{b^*}{C^*})}{k^*} \\ &= \frac{1}{k^*} ln\left(\frac{b^*}{y^*_{start}} - 1\right) + t_{start} \end{aligned}$$

and

$$y^{*'}_{switch} = \frac{k^* b^*}{4}$$

Proof.

$$\begin{aligned} \frac{dy^*_{\text{switch}}2}{dt^2} &= 0 \\ k^*(1 - \frac{2y^*_{\text{switch}}}{b^*}) &= 0 \\ 1 - \frac{2y^*_{\text{switch}}}{b^*} &= 0 \\ y^*_{\text{switch}} &= \frac{b^*}{2} \\ \frac{b^* C^* e^{k^* t_{\text{switch}}}}{b^* + C^* e^{k^* t_{\text{switch}}}} &= \frac{b^*}{2} \\ C^* e^{k^* t_{\text{switch}}} &= b^* \\ ln(e^{k^* t_{\text{switch}}}) &= ln(\frac{b^*}{C^*}) \\ k^* t_{\text{switch}} &= ln(\frac{b^*}{C^*}) \\ t_{\text{switch}} &= \frac{ln(\frac{b^*}{C^*})}{k^*}. \end{aligned}$$

Implement: $C^* = \frac{y^*_{\text{start}}}{(1 - \frac{y^*_{\text{start}}}{b^*}) e^{k^* t_{\text{start}}}}$.
We have:

$$\begin{aligned} t_{\text{switch}} &= \frac{ln(\frac{b^*}{C^*})}{k^*} \\ &= \frac{1}{k^*} ln\left(\frac{b^*(1 - \frac{y^*_{\text{start}}}{b^*}) e^{k^* t_{\text{start}}}}{y^*_{\text{start}}}\right) \\ &= \frac{1}{k^*}\left(ln\left(\frac{(b^* - y^*_{\text{start}})}{y^*_{\text{start}}}\right) + k^* t_{\text{start}}\right) \\ &= \frac{1}{k^*} ln\left(\frac{b^*}{y^*_{\text{start}}} - 1\right) + t_{\text{start}}. \end{aligned}$$

As $y^*_{\text{switch}} = \frac{b^*}{2}$, we also have:

$$\begin{aligned} y^{*'}_{\text{switch}} &= \frac{dy^*_{\text{switch}}}{dt} \\ &= k^* y^*_{\text{switch}}(1 - \frac{y^*_{\text{switch}}}{b^*}) \\ &= k^* \frac{b^*}{2}(1 - \frac{\frac{b^*}{2}}{b^*}) \\ &= \frac{k^* b^*}{4} \end{aligned}$$

□

Proposition 4. *We define the saturation time as the time point where the function reaches* $\hat{y^*} = 0.99b^*$

$$
\begin{aligned}
t_{saturation} &= \frac{1}{k^*} ln\left(\frac{\hat{y^*}b^*}{(b^* - \hat{y^*})C^*}\right) \\
&= \frac{1}{k^*} ln\left(\frac{(\hat{y^*} - y^*_{start})b^*}{(b^* - \hat{y^*})y^*_{start}} + 1\right) + t_{start} \\
&= \frac{1}{k^*} ln\left(99\frac{b^*}{y^*_{start}} - 99\right) + t_{start}.
\end{aligned}
$$

Proof.

$$\hat{y^*} = \frac{b^*C^*e^{k^*t}}{b^* + C^*e^{k^*t}}$$

$$
\begin{aligned}
&\text{Obtain } t_{\text{saturation}}: \\
\hat{y^*}b^* + y^*C^*e^{k^*t} &= b^*C^*e^{k^*t} \\
\hat{y^*}b^* &= e^{k^*t}(b^*C^* - y^*C^*) \\
\frac{\hat{y^*}b^*}{b^*C^* - y^*C^*} &= e^{k^*t} \\
ln\left(\frac{\hat{y^*}b^*}{b^*C^* - y^*C^*}\right) &= ln(e^{k^*t}) \\
ln\left(\frac{\hat{y^*}b^*}{b^*C^* - y^*C^*}\right) &= k^*t \\
t_{\text{saturation}} &= \frac{ln(\frac{\hat{y^*}b^*}{b^*C^* - \hat{y^*}C^*})}{k^*} \\
&\text{Saturation time is where } \hat{y^*} \to b^*
\end{aligned}
$$

Implement: $C^* = \frac{y^*_{\text{start}}}{(1 - \frac{y^*_{\text{start}}}{b^*})e^{k^*t_{\text{start}}}}$.
We have:

$$
\begin{aligned}
t_{\text{saturation}} &= \frac{ln(\frac{\hat{y^*}b^*}{b^*C^* - \hat{y^*}C^*})}{k^*} \\
&= \frac{1}{k^*} ln\left(\frac{\hat{y^*}b^*(1 - \frac{y^*_{\text{start}}}{b^*})e^{k^*t_{\text{start}}}}{(b^* - \hat{y^*})y^*_{\text{start}}}\right) \\
&= \frac{1}{k^*}\left(ln\left(\frac{\hat{y^*}(b^* - y^*_{\text{start}})}{(b^* - \hat{y^*})y^*_{\text{start}})}\right) + k^*t_{\text{start}}\right) \\
&= \frac{1}{k^*} ln\left(\frac{(\hat{y^*} - y^*_{\text{start}})b^*}{y^*_{\text{start}}(b^* - \hat{y^*})} + 1\right) + t_{\text{start}} \; (where\ \hat{y^*} = 0.99b^*) \\
&= \frac{1}{k^*} ln\left(\frac{(0.99b^* - y^*_{\text{start}})b^*}{y^*_{\text{start}}(b^* - 0.99b^*)} + 1\right) + t_{\text{start}} \\
&= \frac{1}{k^*} ln\left(\frac{(0.99b^* - y^*_{\text{start}})b^*}{0.01b^*y^*_{\text{start}}} + 1\right) + t_{\text{start}} \\
&= \frac{1}{k^*} ln\left(\frac{0.99b^* - y^*_{\text{start}}}{0.01y^*_{\text{start}}} + 1\right) + t_{\text{start}} \\
&= \frac{1}{k^*} ln\left(99\frac{b^*}{y^*_{\text{start}}} - 100 + 1\right) + t_{\text{start}} \\
&= \frac{1}{k^*} ln\left(99\frac{b^*}{y^*_{\text{start}}} - 99\right) + t_{\text{start}}
\end{aligned}
$$

□

In a general form, we can define: $\hat{y^*} = m \cdot b^*$ with $m \in [0, 1]$, then we get:

$$
\begin{aligned}
t &= \frac{1}{k^*} ln\left(\frac{(\hat{y^*} - y^*_{\text{start}})b^*}{y^*_{\text{start}}(b^* - \hat{y^*})} + 1\right) + t_{\text{start}} \ (where\ \hat{y^*} = m \cdot b^*) \\
&= \frac{1}{k^*} ln\left(\frac{(mb^* - y^*_{\text{start}})b^*}{y^*_{\text{start}}(b^* - mb^*)} + 1\right) + t_{\text{start}} \\
&= \frac{1}{k^*} ln\left(\frac{mb^* - y^*_{\text{start}}}{(1-m)y^*_{\text{start}}} + 1\right) + t_{\text{start}} \\
&= \frac{1}{k^*} ln\left(\frac{m}{1-m}\frac{b^*}{y^*_{\text{start}}} + \frac{m}{m-1}\right) + t_{\text{start}}
\end{aligned}
$$

By implementing $t_{\text{switch}} = \frac{ln(\frac{b^*}{C^*})}{k^*}$, we can get:

$$
\begin{aligned}
y^*_{\text{switch}} &= \frac{b^* C^* e^{k^* t_{\text{switch}}}}{b^* + C^* e^{k^* t_{\text{switch}}}} \\
&= \frac{b^* C^* e^{k^* \frac{ln(\frac{b^*}{C^*})}{k^*}}}{b^* + C^* e^{k^* \frac{ln(\frac{b^*}{C^*})}{k^*}}} \\
&= \frac{b^* C^* \frac{b^*}{C^*}}{b^* + C^* \frac{b^*}{C^*}} \\
&= \frac{1}{2} \cdot b^*
\end{aligned}
$$

Then, for y^*_{switch} we get t_{switch} for $m = \frac{1}{2}$:

$$
\begin{aligned}
t_{\text{switch}} &= \frac{1}{k^*} ln\left(\frac{m}{1-m}\frac{b^*}{y^*_{\text{start}}} + \frac{m}{m-1}\right) + t_{\text{start}} \\
&= \frac{1}{k^*} ln\left(\frac{0.5}{0.5}\frac{b^*}{y^*_{\text{start}}} + \frac{0.5}{-0.5}\right) + t_{\text{start}} \\
&= \frac{1}{k^*} ln\left(\frac{b^*}{y^*_{\text{start}}} - 1\right) + t_{\text{start}}
\end{aligned}
$$

We could also directly use the general formula and let $m = 0.99$ to have the $t_{\text{saturation}}$ directly:

$$
\begin{aligned}
t_{\text{saturation}} &= \frac{1}{k^*} ln\left(\frac{m}{1-m}\frac{b^*}{y^*_{\text{start}}} + \frac{m}{m-1}\right) + t_{\text{start}} \\
&= \frac{1}{k^*} ln\left(\frac{0.99}{1-0.99}\frac{b^*}{y^*_{\text{start}}} + \frac{0.99}{0.99-1}\right) + t_{\text{start}} \\
&= \frac{1}{k^*} ln\left(99\frac{b^*}{y^*_{\text{start}}} - 99\right) + t_{\text{start}}
\end{aligned}
$$

In the same vein, we could get the minimum time by defining $y_{\text{minimum}} = 10^{-16} \cdot b^*$ We will then get for $m = 10^{-16}$:

$$
\begin{aligned}
t_{\text{minimum}} &= \frac{1}{k^*} ln\left(\frac{10^{-16}}{1-10^{-16}}\frac{b^*}{y^*_{\text{start}}} + \frac{10^{-16}}{10^{-16}-1}\right) + t_{\text{start}} \\
&\approx \frac{1}{k^*} ln\left(\frac{10^{-16}}{1}\frac{b^*}{y^*_{\text{start}}} + \frac{0}{10^{-16}-1}\right) + t_{\text{start}} \\
&\approx \frac{1}{k^*} ln\left(\frac{10^{-16}b^*}{y^*_{\text{start}}}\right) + t_{\text{start}}
\end{aligned}
$$

Proposition 5. *Analytical solution of the generalized form of the logistic ODE.*

Solving the following ODE:

$$\frac{dz}{dt} = k(z-a)(1-\frac{z-a}{b-a})$$

with the starting point of a signal (t_{start}, z_{start}) *and* $z > 0$ *and* $b > z$*, we obtain:*

$$z(t) = \frac{(b-a)Ce^{kt}}{1+Ce^{kt}} + a.$$

Proof. Simplify:

$$\frac{dz}{dt} = \frac{k}{b-a}(z-a)(b-z).$$

Define: $y = z - a$ and $L = b - a$, and substitute in:

$$\frac{dy}{dt} = \frac{k}{b-a}y(b-a-y).$$

Now, the equation is:

$$\frac{dy}{dt} = \frac{k}{L}y(L-y).$$

Separate variables and perform integration:

$$\int \frac{1}{y(L-y)}\,dy = \int \frac{k}{L}\,dt.$$

Use partial fractions:

$$\begin{aligned}
\frac{1}{y(L-y)} &= \frac{1}{L}(\frac{1}{y} + \frac{1}{L-y}) \\
\ln(y) - \ln(L-y) &= kt + c \\
\ln\left(\frac{y}{L-y}\right) &= kt + c \\
\frac{y}{L-y} &= e^{kt+c}
\end{aligned}$$

Define: $C = e^c$.

Find c using the initial point:

$$\begin{aligned}
c &= \ln\left(\frac{y_{\text{start}}}{L-y_{\text{start}}}\right) - kt_{\text{start}} \\
\frac{y}{L-y} &= Ce^{kt} \\
y &= LCe^{kt} - yCe^{kt} \\
y(1+Ce^{kt}) &= LCe^{kt} \\
y(t) &= \frac{LCe^{kt}}{1+Ce^{kt}}.
\end{aligned}$$

Substitute variables L and z:

$$z(t) = \frac{(b-a)Ce^{kt}}{1+Ce^{kt}} + a$$

$$c = \ln\left(\frac{z_{\text{start}} - a}{L - (z_{\text{start}} - a)}\right) - kt_{\text{start}}.$$

□

Proposition 6. *Normalizing y to the range $[R_{min}, R_{max}]$ is equivalent to normalizing z to the range $[R_{min}, R_{max}]$.*

Proof. We define y^* in the normalized range as:

$$y^* = \frac{(y - y_{\text{min}})(R_{\text{max}} - R_{\text{min}})}{y_{\text{max}} - y_{\text{min}}} + R_{\text{min}}$$

with the following relationships:

$$\begin{aligned} y &= z - a; \\ y_{\text{min}} &= z_{\text{min}} - a; \\ y_{\text{max}} &= z_{\text{max}} - a. \end{aligned}$$

Substituting into the expression for y^*:

$$\begin{aligned} y^* &= \frac{((z - a) - (z_{\text{min}} - a))(R_{\text{max}} - R_{\text{min}})}{(z_{\text{max}} - a) - (z_{\text{min}} - a)} + R_{\text{min}} \\ &= \frac{(z - a - z_{\text{min}} + a)(R_{\text{max}} - R_{\text{min}})}{z_{\text{max}} - a - z_{\text{min}} + a} + R_{\text{min}} \\ &= \frac{(z - z_{\text{min}})(R_{\text{max}} - R_{\text{min}})}{z_{\text{max}} - z_{\text{min}}} + R_{\text{min}} \end{aligned}$$

In summary, the normalization cancels out the shift by a, so y^* depends only on z. Thus, normalizing y gives the same result as normalizing z (see **Supplementary Fig. 2**).

□

Proposition 7. *The kinetic parameter k is preserved under translation of the logistic ODE.*

We consider the following logistic ODE:

$$\frac{dy}{dt} = ky\left(1 - \frac{y}{b_y}\right),$$

where $y > 0$ and $b_y > y$.

Given an initial condition $(t_{\text{start}}, y_{\text{start}})$, where y_{start} is the first point in the range of an experimental time-course data sample $y \in [0, b_y]$, we aim to show that this equation is equivalent to a translated version of itself.

Proof. To introduce a translation, we define:

$$z - a = y,$$

where a is a constant shift.
Differentiating both sides with respect to t:

$$\frac{dz}{dt} = \frac{dy}{dt}.$$

Substituting into the original logistic equation:

$$\frac{dz}{dt} = ky\left(1 - \frac{y}{b_y}\right).$$

Now rewrite in terms of z using $y = z - a$, and $b_y = b - a$:

$$\frac{dz}{dt} = k(z - a)\left(1 - \frac{z - a}{b - a}\right).$$

where $z \in [a, b]$.

In summary, shifting by a, i.e., transforming the domain from the observed range $[a, b]$ to the translated range $[0, b_y]$, preserves the logistic form and keeps the parameter k unchanged (see **Supplementary Fig. 2**).

□

Proposition 8. *The kinetic parameter k is preserved under min-max normalization.*

For a given gene or cCRE, let $z(t)$ be a sigmoid function modeling its time-series profile, bounded in the observed data range $[a, b]$ over the time interval $t \in [t_{start}, t_{end}]$:

$$a \leq z(t) \leq b, \quad \forall t \in [t_{start}, t_{end}]$$

We define k as the kinetic parameter in the observed range of the data $[a, b]$, and k^* as the corresponding parameter in the normalized range $[0, b^*]$.

We aim to show that the parameter k is invariant under a linear transformation, specifically under min-max normalization, i.e., $k = k^*$.

Proof. We start with the generalized form of the logistic ODE in the observed range of the data:

$$\frac{dz}{dt} = k(z - a)(1 - \frac{z - a}{b - a})$$

Assuming the normalized data in the range $[0, 1]$ represents a portion of the full sigmoid curve, let $z^* \in [0, b^*]$ be the normalized version of z obtained with min-max normalization, as follows:

$$z^* = \frac{(z - a)(b^* - 0)}{(b - a)}$$

or equivalently:

$$z = \frac{z^*(b - a)}{b^*} + a.$$

Differentiate z with respect to time t:

$$\frac{dz}{dt} = \frac{dz^*}{dt}\frac{b - a}{b^*}$$

Substitute this and the expression for z into the original ODE:

$$\frac{dz^*}{dt}\frac{(b - a)}{b^*} = kz^*\frac{(b - a)}{b^*}(1 - \frac{z^*}{b^*})$$

Simplify and obtain the simplified form of the ODE that applies to the normalized range of the data:

$$\frac{dz^*}{dt} = kz^*(1 - \frac{z^*}{b^*})$$

In summary, the ODE retains the same form in the normalized range, with the same kinetic parameter k (that is, $k^* = k$). Therefore, k is invariant under min-max normalization (see **Supplementary Fig. 2**).

□

Proposition 9. *The key properties of the curve (inflection point and kinetic class) will be preserved under min-max normalization.*

Proof. The inflection point is the point where the second derivative is zero. We have that its first derivative with respect to t is:

$$\frac{d\mathbf{z}}{dt} = \frac{z^*_{\text{max}} - z^*_{\text{min}}}{R_{\text{max}} - R_{\text{min}}} \frac{d\mathbf{z}^*}{dt}$$

The second derivative is defined as:

$$\frac{d^2\mathbf{z}}{dt^2} = \frac{z^*_{\text{max}} - z^*_{\text{min}}}{R_{\text{max}} - R_{\text{min}}} \frac{d^2\mathbf{z}^*}{dt^2}$$

As $\frac{z^*_{\text{max}} - z^*_{\text{min}}}{R_{\text{max}} - R_{\text{min}}}$ is just a constant, we have:

$$\frac{d^2\mathbf{z}}{dt^2} = 0 \quad \text{if and only if} \quad \frac{d^2\mathbf{z}^*}{dt^2} = 0$$

□

Based on this demonstration, we can rescale the normalized curve back to its original range without affecting the switching point; therefore, the inflection point of the normalized and the rescaled curves are the same. In addition, since the kinetic class depends on this point, the kinetic class is also preserved.

Supplementary References

[1] Motulski, H. & Christopoulos, A. *Fitting Models to Biological Data Using Linear and Nonlinear Regression* (Oxford University Press, 2004).
