# Supplementary material for: The chronODE framework for modelling multi-omic time series with ordinary differential equations and machine learning
Source: Nat Commun. 2025 Aug 19;16:7021. doi: 10.1038/s41467-025-61921-9 (PMC12365117; doi:10.1038/s41467-025-61921-9)
Supplement: Supplementary file 2 — Description of Additional Supplementary Files [file 41467_2025_61921_MOESM2_ESM.pdf]

Description of Additional Supplementary Files

File Name: Supplementary Data 1

Description: **Table reporting the kinetic parameters estimated by chronODE for genes and cCREs across three mouse brain regions.** For each element, we report the three logistic ODE parameters (*k*, *a*, and *b*), as well as b^* (*b_starred*), the kinetic parameter from the simplified ODE form. Scaling factors $R_{\min}$ (*R_min*) and $R_{\max}$ (*R_max*), and z_{min} (*z_min*) and z_{max} *(z_max)*, used in data normalization (see Methods, "Data normalization"), are also included. We provide the element's value at the first experimental time point in the real range of the data (z_{start}, denoted as *z_start*). *MSE* refers to the Mean Squared Error of the fit in the normalized space. Kinetic classification details are provided: kinetic class, switching time, saturation time, and minimum time (see Methods, section "2.4 Kinetic Classification", and Supplementary Note 1).
