# Supplementary material for: The chronODE framework for modelling multi-omic time series with ordinary differential equations and machine learning
Source: Nat Commun. 2025 Aug 19;16:7021. doi: 10.1038/s41467-025-61921-9 (PMC12365117; doi:10.1038/s41467-025-61921-9)
Supplement: Supplementary file 4 — Reporting Summary [file 41467_2025_61921_MOESM4_ESM.pdf]

nature portfolio

Corresponding author(s): Mark Gerstein
Last updated by author(s): 06/09/2025

Reporting Summary

Nature Portfolio wishes to improve the reproducibility of the work that we publish. This form provides structure for consistency and transparency in reporting. For further information on Nature Portfolio policies, see our Editorial Policies and the Editorial Policy Checklist.

Statistics

For all statistical analyses, confirm that the following items are present in the figure legend, table legend, main text, or Methods section.

n/a	Confirmed	
☐	☒	The exact sample size (n) for each experimental group/condition, given as a discrete number and unit of measurement
☐	☒	A statement on whether measurements were taken from distinct samples or whether the same sample was measured repeatedly
☐	☒	The statistical test(s) used AND whether they are one- or two-sided<br>*Only common tests should be described solely by name; describe more complex techniques in the Methods section.*
☒	☐	A description of all covariates tested
☐	☒	A description of any assumptions or corrections, such as tests of normality and adjustment for multiple comparisons
☐	☒	A full description of the statistical parameters including central tendency (e.g. means) or other basic estimates (e.g. regression coefficient) AND variation (e.g. standard deviation) or associated estimates of uncertainty (e.g. confidence intervals)
☐	☒	For null hypothesis testing, the test statistic (e.g. F, t, r) with confidence intervals, effect sizes, degrees of freedom and P value noted<br>*Give P values as exact values whenever suitable.*
☒	☐	For Bayesian analysis, information on the choice of priors and Markov chain Monte Carlo settings
☒	☐	For hierarchical and complex designs, identification of the appropriate level for tests and full reporting of outcomes
☐	☒	Estimates of effect sizes (e.g. Cohen's d, Pearson's r), indicating how they were calculated

Our web collection on statistics for biologists contains articles on many of the points above.

Software and code

Policy information about availability of computer code

Data collection	ENCODE data (RNA-seq, ATAC-seq, DNase-seq, catalog of cCREs) were downloaded from the ENCODE portal using wget.<br>Single-cell data from Qiu et al., 2024, was downloaded from https://omg.gs.washington.edu/jax/public/download.html using wget.
Data analysis	The analyses were performed using R (v4.2.3) or Python (v3.9.16). The chronODE pipeline for monotonic fitting is written in Nextflow DSL2 (v23.04.1). A yml file listing the specific software dependencies is available on both the GitHub and Zenodo repositories. The software is available at https://github.com/gersteinlab/chronODE (DOI: 10.5281/zenodo.15574976). A detailed description of the software employed is provided in the Methods and the Supplementary Note 1.

For manuscripts utilizing custom algorithms or software that are central to the research but not yet described in published literature, software must be made available to editors and reviewers. We strongly encourage code deposition in a community repository (e.g. GitHub). See the Nature Portfolio guidelines for submitting code & software for further information.

Data

Policy information about availability of data

All manuscripts must include a data availability statement. This statement should provide the following information, where applicable:

- Accession codes, unique identifiers, or web links for publicly available datasets
- A description of any restrictions on data availability
- For clinical datasets or third party data, please ensure that the statement adheres to our policy

All the data employed in this study are publicly available. ENCODE data were obtained from the ENCODE Portal (www.encodeproject.org, accession numbers of files are provided in Supplementary Tables 1-3). The catalog of mouse cCREs was downloaded from the ENCODE portal (https://www.encodeproject.org/annotations/ENCSR412JPD/) (Moore et al., 2024 bioRxiv). The single-cell RNA-seq data were obtained from https://omg.gs.washington.edu/jax/public/download.html (see "gene expression across timepoints") (Qiu et al., 2024 Nature). The processed data resulting from the kinetic classification of genes and cCREs across the three brain regions are available in Supplementary Data 1. Source data are provided with this paper.

Research involving human participants, their data, or biological material

Policy information about studies with human participants or human data. See also policy information about sex, gender (identity/presentation), and sexual orientation and race, ethnicity and racism.

Reporting on sex and gender	Not applicable
Reporting on race, ethnicity, or other socially relevant groupings	Not applicable
Population characteristics	Not applicable
Recruitment	Not applicable
Ethics oversight	Not applicable

Note that full information on the approval of the study protocol must also be provided in the manuscript.

Field-specific reporting

Please select the one below that is the best fit for your research. If you are not sure, read the appropriate sections before making your selection.

☒ Life sciences ☐ Behavioural & social sciences ☐ Ecological, evolutionary & environmental sciences

For a reference copy of the document with all sections, see nature.com/documents/nr-reporting-summary-flat.pdf

Life sciences study design

All studies must disclose on these points even when the disclosure is negative.

Sample size	No statistical methods were applied to predetermine sample sizes. All the sample sizes of sequencing data were based on standard sequencing analysis practices and were determined to be suitable for statistical analyses.
Data exclusions	No data were excluded from the analysis.
Replication	Multiple replicates were taken into account wherever available. Correlation between replicates was assessed by the ENCODE Consortium. Significant changes in time-series profiles of chromatin accessibility and gene expression were detected by taking into account both biological replicates (i.e. replicates are handled internally by the maSigPro algorithm).
Randomization	Randomization is not relevant in this study because no comparisons between experimental groups were made.
Blinding	Blinding is not relevant in this study because all metrics were derived from absolute quantitative methods without human subjectivity.

Reporting for specific materials, systems and methods

We require information from authors about some types of materials, experimental systems and methods used in many studies. Here, indicate whether each material, system or method listed is relevant to your study. If you are not sure if a list item applies to your research, read the appropriate section before selecting a response.

Materials & experimental systems

n/a	Involved in the study
☒	☐ Antibodies
☒	☐ Eukaryotic cell lines
☒	☐ Palaeontology and archaeology
☒	☐ Animals and other organisms
☒	☐ Clinical data
☒	☐ Dual use research of concern
☒	☐ Plants

Methods

n/a	Involved in the study
☒	☐ ChIP-seq
☒	☐ Flow cytometry
☒	☐ MRI-based neuroimaging

Plants

Seed stocks	Not applicable
Novel plant genotypes	Not applicable
Authentication	Not applicable
